# Analysis of survival and prognostic factors in appendix adenocarcinoma and mucinous carcinoma

**DOI:** 10.1007/s13304-025-02103-6

**Published:** 2025-01-17

**Authors:** Bilal Turan, Ahmet Necati Sanli, Serdar Acar

**Affiliations:** 1 Suleyman Demirel Univercity, Isparta, Turkey; 2 Gaziantep Abdülkadir Yüksel, State Hospital, Gaziantep, Turkey

**Keywords:** Adenocarcinoma, Appendix, Mucinous carcinoma

## Abstract

This study aimed to compare mucinous carcinoma and adenocarcinoma of the appendix in terms of survival and investigate the risk factors influencing survival. The data for this study were retrieved from the SEER database (SEER Research Plus 17 registries). Patients diagnosed with appendix cancer between 2004 and 2019 were included. Demographic data, such as age, gender, marital status, and year of diagnosis, along with oncological variables like stage, surgery, chemotherapy, radiotherapy, and survival time, were extracted from the SEER database. Pathological subtypes were classified as adenocarcinoma (AC) and mucinous adenocarcinoma (MAC) based on the College of American Pathologists guidelines. Patients with other pathological subtypes or missing data were excluded from the study. This study included 4524 patients, with 2118 (46.8%) classified as AC and 2406 (53.2%) as MAC. There was no significant difference in mean age between AC and MAC groups (63.22 ± 14.30 vs. 59.46 ± 14.07, *p* = 0.483). AC was more common in males, while MAC was more prevalent in females (46.8% vs. 53.2%; 55.6% vs. 44.4%, *p* < 0.001, respectively). Married status was high in both groups (*p* = 0.001). While no difference was found in white race distribution, the black race was more prevalent in the AC group (57.1% vs. 42.9%, *p* < 0.001). Grade 1 tumors were more frequent in the AC group, whereas Grades 2 and 3 were more common in the MAC group (*p* < 0.001). Stages 1, 2, and 3 were more prevalent in the AC group, while the majority of MAC cases were at Stage 4. Surgery rates were higher in the AC group (98.6% vs. 96.4%, *p* < 0.001). Chemotherapy was used more frequently in the MAC group (50.9% vs. 40.6%, *p* < 0.001), while radiotherapy rates were similar in both groups (*p* = 0.498). The mean follow-up period was 55.70 ± 47.2 months. Five- and ten-year survival rates for the MAC group were 64.4% and 50.2%, respectively, higher than the AC group’s rates of 54.2% and 39.7% (*p* < 0.001). The overall risk of mortality was 1.4 times higher in the AC group compared to the MAC group (*p* < 0.001, HR: 1.377 [CI 95% 1.259–1.507]). While adenocarcinomas and mucinous adenocarcinomas have similar incidences, non-metastatic adenocarcinomas were more frequently observed. In contrast, mucinous adenocarcinomas often exhibited distant metastases. Nevertheless, the survival rate was higher in mucinous adenocarcinomas.

## Introductıon

Primary appendiceal cancers are rare, with an incidence of approximately 1.2 cases per 100,000 individuals annually in the United States. They are most commonly discovered incidentally during pathological examination of surgical specimens following appendectomy for acute appendicitis. Rarely, they may also be identified incidentally during abdominal imaging, colonoscopy, or surgery. Histologically and biologically, they are divided into distinct subtypes. Historically, the classification of these tumors has been confusing due to varying terminology. In general, they can be categorized into four main subtypes:

1. Colonic-type adenocarcinoma
2. Mucinous neoplasm
3. Goblet cell carcinoma
4. Neuroendocrine neoplasm

Signet-ring cells are considered a histological feature that can be observed in colonic-type adenocarcinomas and mucinous neoplasms, rather than a distinct subtype. Proper classification of these subtypes is essential as staging and management of appendiceal tumors depend on them. [1–3].

This study aims to provide an overview of the epidemiology, grading, staging, management, and prognosis of these rare neoplasms, focusing on adenocarcinomas and mucinous neoplasms.

## Materıals and methods

### Data source and study population

In the study, the data of patients diagnosed with appendix cancer in the years 2004–2019 were extracted using the SEER 17 Registries Research Plus data covering 26.5% of the American population. Data were obtained from the SEER*Stat program. Patients aged 18 years, with only one primary cancer and Histologic ICD-O-3 codes: “8140/3–8221/2”–Adenocarcinoma, “8470/3–8481-3”- Mucinous adenocarcinoma were included. Age at diagnosis (< 50, ≥ 50), marital status (married, others) and race (white, black, other) as demographic variables, grade (I, II, III), stage (I, II, III, IV), surgery (performed, not performed), radiotherapy (performed, not performed), and chemotherapy (performed, not performed). Survival time, vital status and cause of death were used as oncologic results. Patients with missing data and whose stage was 0 were also excluded from the cohort.

As this study utilized de-identified data from the SEER database, which is publicly available and maintains patient anonymity, ethical approval and informed consent were not required.

### Statistical analysis

Statistical analysis was performed using SPSS for Windows (version 22.0, SPSS Inc., Chicago, IL, USA). Kolmogorov–Smirnov test and box plot graphs were used to assess the study data conformity to the normal distribution of the variables, together with descriptive statistical methods (mean, standard deviation). Independent samples T test was used for comparisons between groups of normally distributed variables. Pearson chi-square test was used to compare qualitative data. Based on who was still alive at the end of the study or at the last follow-up, overall survival (OS) was estimated. For those who were alive at the end of the study period, died due to another cause, or were still alive at their last follow-up and died of appendix cancer, cancer-specific survival (BCSS) was computed. The findings of survival analyses were evaluated using Kaplan–Meier analysis and the log-rank test. For both univariate and multivariate analyses, Cox regression analysis was performed. Significance was evaluated at the *p* < 0.05 level.

## Results

### Clinicopathologic characteristics

A total of 4,524 patients met the inclusion criteria, comprising 2,117 cases of adenocarcinoma (46.8%) and 2,406 cases of mucinous carcinoma (53.2%). The AC group included significantly older patients compared to the MAC group (63.22 ± 14.30 vs. 59.46 ± 14.07, *p* < 0.001). AC was more commonly seen in males, while MAC was more frequent in females (46.8% vs. 53.2%; 55.6% vs. 44.4%, *p* < 0.001). The most common ethnic group in both groups was white (AC: 80.5%; MAC: 82.2%) although the proportion of black patients was higher in the AC group (12.9% vs. 8.6%). Marital status showed that both groups had a higher proportion of married patients compared to unmarried ones; however, the proportion of married individuals was significantly higher in the MAC group (*p* = 0.001). [Table 1].

**Table 1:** Patients characteristics

Characteristics		Adenocarcinoma (n:2118)		Mucinous carcinoma (n:2406)		
		*N*	%	*N*	%	*P*
Age at diagnosis	Median ± SD	63.22 ± 14.30		59.46 ± 14.07		< 0.001a
	≤ 50	406	19.2	621	25.8	< 0.001b
	> 50	1712	80.8	1785	74.2	< 0.001b
Gender	Female	991	46.8	1338	55.6	< 0.001b
	Male	1127	53.2	1068	44.4	
Race	White	1705	80.5	1978	*82.2*	< 0.001b
	Black	274	12.9	480	8.6	
	Others	139	6.6	361	9.2	
Marital status	Married	1307	61.7	1594	66.3	0.001
	Others	811	38.3	812	33.7	
Grade	I	385	18.2	1102	45.8	< 0.001b
	II	1243	58.7	1043	43.3	
	III	490	23.1	261	10.8	
Stage	0	31	1.5	22	0.9	< 0.001b
	I	397	18.7	199	8.3	
	II	867	40.9	825	34.3	
	III	411	19.4	225	9.4	
	IV	412	19.5	1135	47.2	
Surgery	Performed	2089	98.6	2319	96.4	< 0.001b
	Not performed	29	1.4	87	3.6	
Radiotherapy	Yes	48	2.3	62.6	2.6	0.499b
	No	2070	97.7	2344	97.4	
Chemotherapy	Yes	860	40.6	1226	51	< 0.001b
	No	1258	59.4	1110	49	
Survival time (month)	Median ± SD (Min–Max)	55.62 ± 47.07 (0–119)				

Statistical differences in tumor grade between AC and MAC groups were notable. The MAC group predominantly exhibited lower-grade tumors (Grade I: 45.8%), while higher-grade tumors (Grade III: 23.1%) were more prevalent in the AC group (*p* < 0.001). Regarding cancer stage, significant differences were observed between the groups. Stage I and II cases were more frequently observed in the AC group (Stage I: 18.7%; Stage II: 40.9%), while Stage IV cases were predominant in the MAC group (47.2% vs. 19.5%) (*p* < 0.001). Overall, MAC cases were generally diagnosed at advanced stages, whereas AC cases were diagnosed at earlier stages. [Table 1].

Surgical treatment was widespread in both groups, but the AC group had a significantly higher surgical intervention rate (98.6% vs. 96.4%, *p* < 0.001). Chemotherapy was used more frequently in the MAC group (51% vs. 40.6%, *p* < 0.001), while radiotherapy showed no significant difference between groups (*p* = 0.498). [Table 1].

### Cancer-specific survival

**Fig. 1:**
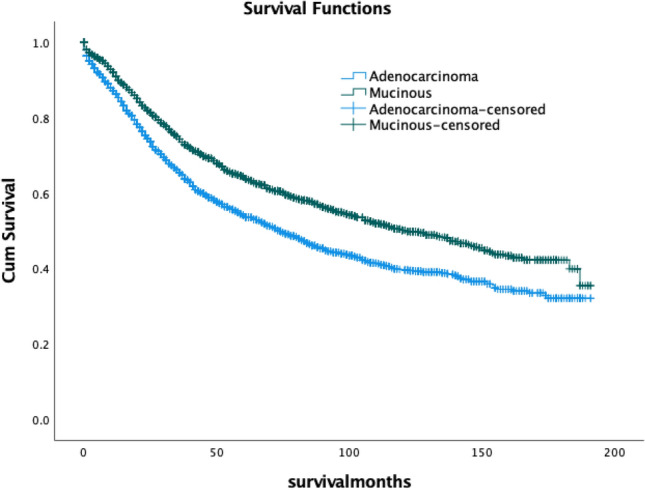
Cancer-specific survival (CSS)

The mean follow-up time was 55.70 ± 47.2 months. While the overall survival (OS) rates for AC and MAC were similar (32.1% vs. 35.5%), the 5-year and 10-year survival rates were worse for adenocarcinoma. The 5-year survival rates were 54.1% for AC and 64.4% for MAC, and the 10-year survival rates were 39.6% for AC and 50.2% for MAC. MAC patients had significantly better survival rates compared to AC patients (*p* < 0.001, HR: 1.377 [95% CI 1.259–1.507]). Although both groups exhibited a cumulative decline in survival over time, the survival curve for MAC consistently remained superior to that of AC. [Fig. 1]. [Table 2].

**Table 2:** All patients cox regression analysis

		Univariate				Multivariate			
		*p*	HR	95% CI for HR				95% CI for HR	
				Lower	Upper	*p*	HR	Lower	Upper
Group	Mucinous		Reference				Reference		
	Adenocarcinoma	< 0.001	1.379	1.260	1.509	< 0.001	1.503	1.354	1.669
Age	** ≤ 50**						Reference		
	** > 50**	< 0.001	1.540	1.370	1.731	< 0.001	1.583	1.406	1.782
Gender	Female		Reference				Reference		
	Male	< 0.001	1.124	1.028	1.230	< 0.001	1.249	1.139	1.370
Marital status	Married		Reference				Reference		
	Other	< 0.001	1.254	1.144	1.375	< .001	1.315	1.197	1.446
Race		0.049							
	White	0.043	Reference			0.082	Reference		
	Black	0.015	1.190	1.034	1.371	0.031	1.171	1.014	1.352
	Other	0.693	0.966	0.814	1.146	0.711	0.968	0.816	1.149
Grade		< 0.001							
	I	< 0.001	Reference			< 0.001	Reference		
	II	< 0.001	1.565	1.399	1.750	< 0.001	1.526	1.358	1.715
	III	< 0.001	3.246	2.854	3.693	< 0.001	2.842	2.472	3.267
Stage		< 0.001				< 0,001	2.529	2.356	2.714
	0	< 0.001	Reference			< .001	Reference		
	I	0.835	1.058	1.523	1.679	.998	1.001	.589	1.701
	II	0.097	1.544			.090	1.559	.933	2.607
	III	< 0.001	2.732	1.165	1.262	< .001	2.492	1.476	4.207
	IV	< 0.001	3.083	1.850	5.136	< .001	4.304	2.566	7.221
Surgery	Performed		Reference				Reference		
	Not performed	< 0.001	2.511	2.009	3.139	< 0.001	1.997	1.589	2.509
Chemotherapy	Yes		Reference				Reference		
	No	0.001	0.860	0.785	0.943	< 0.001	1.317	1.189	1.460
Radiotherapy	Yes		Reference				Reference		
	No	0.234	0.857	0.664	1.105	0.003	.679	.525	.879

### Cox regression analysis for all patients

To further evaluate the effects of grade and stage for AC and MAC, a multivariate model was created using “unknown grade” as a separate category, and hazard ratios (HRs) were calculated. Even after removing or re-categorizing histologic grades in the multivariate model, the findings remained consistent. [Table 2].

The AC group had a higher survival risk compared to the MAC group, with an HR of 1.503 (95% CI 1.354–1.669, *p* < 0.001).

Patients aged over 50 years had a significantly higher HR compared to those aged 50 years or younger (HR: 1.583, 95% CI 1.406–1.782, *p* < 0.001). Males exhibited a higher HR than females (HR: 1.249, 95% CI 1.139–1.370, *p* < 0.001). Unmarried patients had a higher HR compared to married ones (HR: 1.315, 95% CI 1.197–1.446, *p* < 0.001). There were no statistically significant differences in HRs across racial groups in either univariate or multivariate analyses (*p* > 0.05).

Tumor grade and stage were critical factors influencing survival. Grade III tumors had the highest risk (HR: 2.842, *p* < 0.001), and Stage IV cases had dramatically increased survival risks (HR: 4.304, *p* < 0.001). Patients who did not undergo surgery were significantly more at risk compared to those who underwent surgery (HR: 1.997, 95% CI 1.589–2.509, *p* < 0.001). Chemotherapy was associated with improved survival, while its absence increased risk (HR: 1.317, *p* < 0.001). Similarly, the lack of radiotherapy increased survival risk (HR: 0.679, *p* = 0.003).

MAC demonstrated a better survival profile than AC. The mortality risk for AC was 1.4 times higher than for MAC (HR: 1.379 [95% CI 1.260–1.509], *p* < 0.001). Although MAC was frequently diagnosed at advanced stages, it exhibited longer survival compared to AC.

## Dıscussıon

This study extensively evaluated the factors influencing survival in patients diagnosed with AC and MAC. The results revealed significant clinical, demographic, and biological differences between the two tumor subtypes. These distinctions highlight the need for tailored patient management and treatment approaches based on tumor subtypes.

Compared to AC, MAC is often diagnosed at a younger age and is more common in females. This suggests that MAC may have a distinct biological profile and a less aggressive clinical course. Conversely, AC is more frequently observed in older males. Similar findings have been reported in the literature, where MAC is described as predominantly affecting younger populations and females. Recognizing these differences is crucial for diagnosis and treatment planning. [4, 5].

Both AC and MAC showed that patients over the age of 50 had a significantly higher risk of mortality compared to younger patients. Univariate and multivariate analyses revealed that patients aged above 50 had considerably higher hazard ratios (HR) compared to those aged 50 and below (*p* < 0.001). This underscores the significant impact of age on the prognosis of appendiceal cancers. Our analysis also indicated that AC patients were significantly older than MAC patients, with a higher prevalence of AC observed in individuals above 50 years of age. These findings suggest that further research is necessary to explore the underlying causes of these age-related differences and their effects on prognosis. Literature corroborates that AC typically presents in males aged 62–65 on average, while MAC has a mean presentation age of 60, with a slight female predominance but no definitive gender bias [5–7].

Although statistical significance was not observed, black race was more frequently associated with the adenocarcinoma group compared to the mucinous carcinoma group. The influence of race on appendiceal cancer prognosis remains unclear; however, further studies with larger sample sizes and molecular-level investigations are needed. In both univariate and multivariate analyses, no significant relationship was found between race and outcomes (*p* > 0.05) [8].

There are no studies in the literature addressing the impact of marital status on appendiceal cancers. Our analysis revealed that being married had a positive impact on the survival of patients with appendiceal cancer. Within the groups, the rate of married individuals was statistically higher in the MAC group compared to the AC group. Both AC and MAC showed that unmarried patients had a significantly higher mortality risk compared to married patients (*p* < 0.001, *p* < 0.003, respectively). These findings suggest that social support and marital factors may play a role in patient outcomes.

Survival analysis showed worse prognoses for AC compared to MAC. Kaplan–Meier curves and multivariate analyses indicated significantly higher survival risks for AC, suggesting a more aggressive biological behavior and poorer outcomes. Despite being diagnosed at more advanced stages, MAC demonstrated better survival rates, supporting its distinct biological characteristics [4, 9].

Tumor grade and stage have a significant impact on survival for both tumor types. Tumors with lower grades and earlier stages were associated with better survival rates. Notably, the survival risk increased dramatically in patients with Grade III and Stage IV tumors. Higher tumor grades (Grade II and III) were significantly associated with higher hazard ratios (HR) compared to Grade I in both univariate and multivariate analyses (*p* < 0.001). This underscores the importance of tumor histologic grade in determining patient prognosis. These findings further emphasize the critical role of early diagnosis and treatment. Literature also indicates that patients diagnosed at advanced stages tend to have worse prognoses and limited responses to treatment [10–12].

However, there are notable differences in histologic grades between AC and MAC. Grade I tumors were more common in MAC, whereas Grade II and III tumors were more prevalent in AC. This suggests that histologic grade is a significant factor in predicting prognosis for appendiceal cancer. Additionally, histologic grade appears to be a stronger prognostic factor for AC than for MAC. The mortality risk associated with AC was found to be higher than that of MAC. These results emphasize the critical role of histologic grade in predicting patient prognosis [13–15].

The findings indicate that cancer stage significantly affects survival outcomes for appendiceal cancer patients. For both adenocarcinoma and mucinous carcinoma, higher cancer stages (Stages II, III, and IV) were associated with increased mortality risk (*p* < 0.001). Although there was no difference in Stage 0, Stages I and II were more frequently observed in AC, while Stage IV was more common in MAC. The likelihood of presenting with Stage IV disease was 2.5 times higher in MAC compared to AC (47% vs. 19%, respectively). While this finding aligns with previous reports in the literature, our analysis showed an even higher rate. Stage III disease was relatively rare in MAC (9%) but more pronounced in AC (19.4%). These findings, consistent with the literature, suggest that MAC has a higher tendency for peritoneal metastasis rather than lymphatic metastasis. These results highlight the importance of cancer stage in treatment planning and prognosis prediction [12, 14].

The positive effects of surgery and chemotherapy on survival were clearly demonstrated in this study. Surgical treatment emerged as the most significant factor in improving survival for both groups. The rate of surgical intervention was significantly higher in the AC group compared to the MAC group (*p* < 0.001). For both AC and MAC, not undergoing surgery was associated with a significantly higher risk of mortality (*p* < 0.001).

Furthermore, chemotherapy played a particularly beneficial role in improving survival in the adenocarcinoma group. On the other hand, the impact of radiotherapy on survival was found to be more limited, a finding consistent with the literature. The optimal combination of treatment modalities, especially for more aggressive tumor types like adenocarcinoma, has the potential to improve patient prognosis [1, 2, 10, 16–18].

This study demonstrated significant clinical and biological differences between AC and MAC, highlighting the need for subtype-specific management strategies. Although MAC patients exhibited better survival rates, they were generally diagnosed at more advanced stages. These findings emphasize the importance of individualized treatment planning based on tumor subtype. Surgery and chemotherapy were clearly identified as the most significant factors positively influencing survival. Prognostic factors, such as age, gender, and tumor characteristics, should be carefully considered in clinical decision-making. These results offer valuable insights for improving patient outcomes in clinical practice. Nevertheless, prospective studies involving larger patient populations are necessary to enhance the generalizability of these findings.

## Data Availability

All data generated or analyzed during this study are included in this published article [and its supplementary information files].
